# MK2 Promotes the Development and Progression of Pancreatic Neuroendocrine Tumors Mediated by Macrophages and Metabolomic Factors

**DOI:** 10.3390/ijms232113561

**Published:** 2022-11-05

**Authors:** Damian Jacenik, Eric J. Lebish, Ellen J. Beswick

**Affiliations:** 1 Department of Cytobiochemistry, Faculty of Biology and Environmental Protection, University of Lodz, 90-236 Lodz, Poland; 2 Division of Gastroenterology, Hepatology and Nutrition, Department of Internal Medicine, University of Utah, Salt Lake City, UT 84132, USA

**Keywords:** mitogen-activated protein kinase-activated protein kinase 2, MAPKAPK2, MK2, pancreas, pancreatic neuroendocrine tumor, neuroendocrine tumor

## Abstract

Cases of pancreatic neuroendocrine tumors (PNETs) are growing in number, and new treatment options are needed in order to improve patient outcomes. The mitogen-activated protein kinase-activated protein kinase 2 (MK2) is a crucial regulator of cytokine/chemokine production. The significance of MK2 expression and signaling pathway mediated by MK2 in PNETs has not been investigated. To characterize the impact of MK2 on PNET growth, we used the *RipTag2* transgenic murine model of PNETs, and we developed a primary PNET cell line for both in vitro and in vivo studies. In the transgenic murine model of PNETs, we found that MK2 inhibition improves survival of mice and prevents PNET progression. MK2 blockade abolished cytokine/chemokine production, which was related to macrophage function. A role for MK2 in the regulation of metabolic factor secretion in PNETs was identified, making this the first study to identify a potential role for the MK2 pathway in regulation of tumor metabolism. Moreover, using an in vitro approach and allograft model of PNETs, we were able to show that macrophages with MK2 depletion exhibit increased cytotoxicity against PNET cells and substantially decreased production of pro-inflammatory cytokines and chemokines, as well as metabolic factors. Taken together, our work identifies MK2 as a potent driver of immune response and metabolic effectors in PNETs, suggesting it is a potential therapeutic target for patients with PNETs.

## 1. Introduction

Pancreatic neuroendocrine tumors (PNETs) are formed from the islet cells of the pancreas and are growing in number [1]. PNETs are a heterogenous type of tumor that are difficult to treat, which is related to the lack of specific pathways responsible for its progression [2]. From the clinical point of view, PNETs are classified as functional and non-functional PNETs, based on the presence or absence of hormone hypersecretion from PNET cells. Non-functional PNETs are often asymptomatic or minimally symptomatic and the interpretation of functional PNET symptoms is often ambiguous, which is related to the detection of PNETs in advanced stages [3]. The functional PNETs secrete a wide spectrum of metabolic factors such as insulin, somatostatin and glucagon, which is challenging for PNET diagnosis. While surgical intervention and/or some targeted therapies are available for patients with PNETs, more effective treatments are needed to improve patient outcomes [4,5,6]. Limited efforts have been made to explore treatment approaches to prevent both the progression of PNETs and metabolic symptoms in patients with PNETs. Thus, the development of novel treatment strategies for patients with PNETs is clearly needed.

Here, we examined the mitogen-activated protein kinase-activated protein kinase 2 (MK2) as a potent regulator of tumorigenesis. MK2 is a stress-activated serine/threonine-protein kinase involved in cytokines production, chromatin remodeling, DNA damage response, cell cycle and transcriptional regulation [7,8]. The clinical relevance of MK2 activity has been demonstrated in some cancer types such as breast, colon and pancreas cancers, as well as multiple myeloma [9,10,11,12,13]. Our group and others have highlighted an essential role of MK2 in the regulation of tumor-promoting immune responses through cytokine and chemokine production in cancers [11,14,15]. According to experimental data, MK2 knockout (KO) mice show increased resistance to LPS and reduced levels of numerous pro-inflammatory cytokines such as interleukin (IL)-1β, IL-6, interferon-γ (IFN-γ) and tumor necrosis factor-α (TNF-α) compared to wild type (WT) animals [14]. Moreover, it was documented that MK2 modulates not only the production of cytokines, but also chemokines such as monocyte chemoattractant protein-1 (MCP-1), macrophage inflammatory protein-1 (MIP-1) α and β production, which promotes the progression of colon cancer [15]. Nevertheless, the significance of MK2 as a regulator of the development and progression of PNETs has not been examined.

We hypothesized that MK2 expression and signaling is a crucial regulator of PNET progression by affecting pro-tumorigenic immune responses. To investigate the therapeutic potential of MK2 expression and activity on the progression and modulation of metabolomic factors in PNETs, we used MK2 inhibitors (MK2i), WT and MK2 KO bone marrow-derived macrophages (BMMs), a transgenic mice model of PNETs and a novel primary PNET cell-derived allograft tumor model. Moreover, in vitro analyses using BMMs and primary PNET cells were employed to examine the role of MK2 in macrophage function in the PNET microenvironment. Since PNETs are metabolically active tumors, we found a novel role for MK2 function in the context of the PNET metabolome. Our study shows the pro-tumorigenic action of MK2 in PNETs modulated at least partially by macrophages and the potent ability of MK2 inhibition to prevent the progression of PNETs, affecting not only cytokine and chemokine production, but also a wide spectrum of metabolomic factors. Thus, the potential of MK2 inhibitors as a novel therapeutic for PNETs should be investigated.

## 2. Results

### 2.1. Inhibition of MK2 Improves Survival and Prevents the Progression of Pancreatic Neuroendocrine Tumors

**Figure 1:**
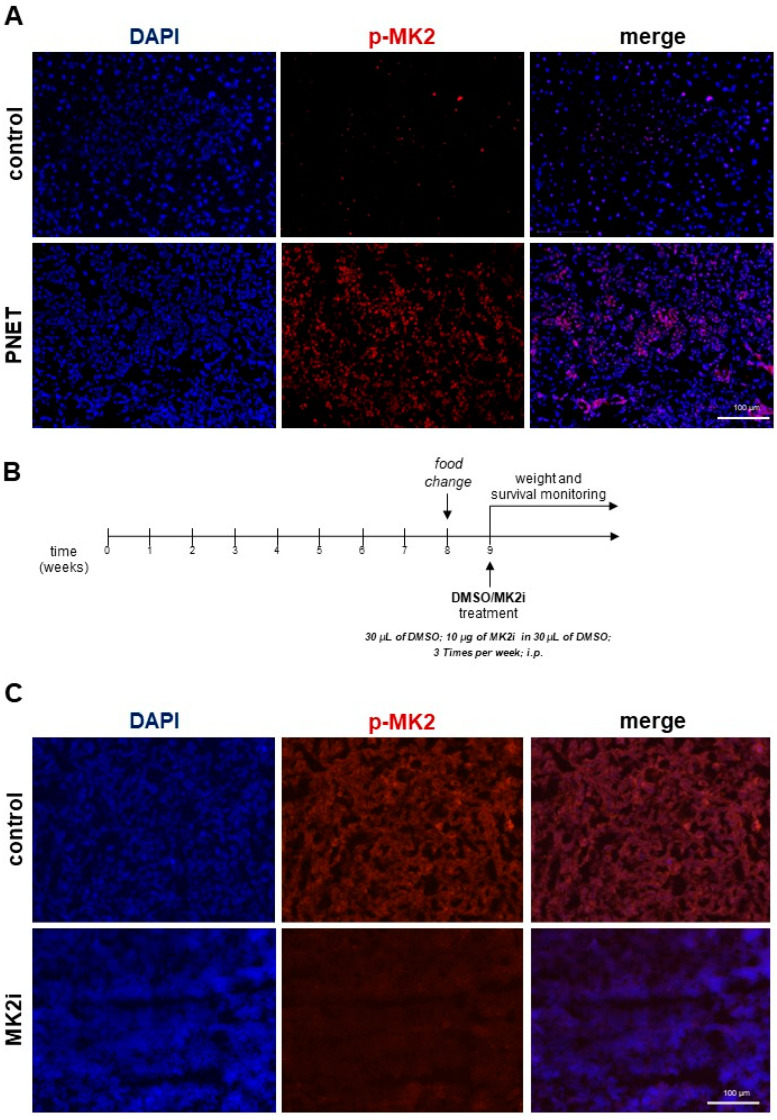

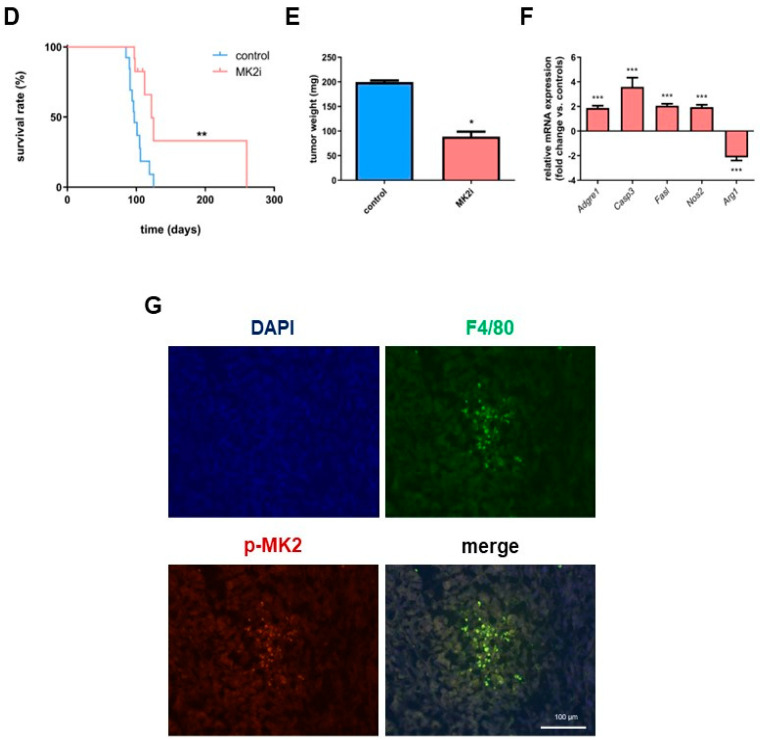
**MK2 is activated in mouse PNETs, and its inhibition improves survival time and suppresses tumor growth in a PNET model**. Control pancreas and PNETs were stained for phospho-MK2 (**A**), where was highly expressed as shown by a representative image. The *RipTag2* mice were treated as shown in a timeline (**B**) where the sequence of procedures and treatments in the transgenic *RipTag2* mice model of PNETs were presented. Furthermore, immunofluorescence staining (**C**) and Kaplan–Meier curve (**D**) show decreased activity of MK2 and increased survival time in MK2 inhibitor-treated mice (MK2i, red), and the weight of PNETs (**E**) were decreased in mice treated with MK2 inhibitor (n = 14 in each group). In PNETs, the gene expression (**F**) of *Adgre1*, *Casp3*, *Fasl* and *Nos2* are increased while *Arg1* is decreased in mice treated with MK2 inhibitor, (n = 6 in each group). PNETs were stained for phospho-MK2 and F4/80 where co-expression of MK2 and macrophage marker was shown by a representative image (**G**). Data are presented as means  ±  SEM; * *p* <  0.05, ** *p* <  0.01, *** *p* <  0.001 vs. control.

In order to understand the significance of MK2 in the progression of PNETs, we first examined MK2 activity in tumors from the transgenic mouse model of PNETs, using Tg(RIP1-Tag)2Dh (*RipTag2*) mice where PNETs develop spontaneously from hyperplasia to carcinoma. A representative image suggests that PNET tumors have high levels of phospho-MK2 compared to a normal pancreas (Figure 1A). We then established a treatment approach for the transgenic *RipTag2* mice to examine MK2 blockade. These mice typically develop tumors by week 8, so for these studies treatments and examination of weight and survival time began in week 9 as depicted in Figure 1B. We found this treatment approach to be sufficient to inhibit expression of phospho-MK2 in mouse tumors as shown in Figure 1C. Overall, inhibition of MK2 was found to significantly improve the survival rate of the transgenic *RipTag2* mice compared to control animals (Figure 1D). Moreover, we found that inhibition of MK2 significantly decreased PNETs by approximately 50% (Figure 1E). Real-time PCR analysis of tumors indicated up to a four-fold increase in the expression of apoptotic genes such as *Casp3* and *Fasl* in PNETs obtained from *RipTag2* mice treated with MK2i compared to control animals (Figure 1F). Furthermore, in tumors obtained from animals treated with MK2i, gene expression analysis showed a significantly increased expression of *Adgre1* in relation to controls, suggesting a role for macrophages in PNETs related to MK2 activity (Figure 1F). We also found and increase in *Nos2* and a decrease in *Arg1* gene expression, suggesting an increase in anti-tumorigenic macrophages and a decrease in pro-tumorigenic macrophages. Of note, tumors were also stained for F4/80 and phospho-MK2 indicating that MK2 activity is not only in tumor cells, but also in macrophages (Figure 1G).

### 2.2. Inhibition of MK2 Affects Production and Secretion of Pro-Tumorigenic Macrophage-Related Cytokines and Chemokines in Pancreatic Neuroendocrine Tumors

**Figure 2:**
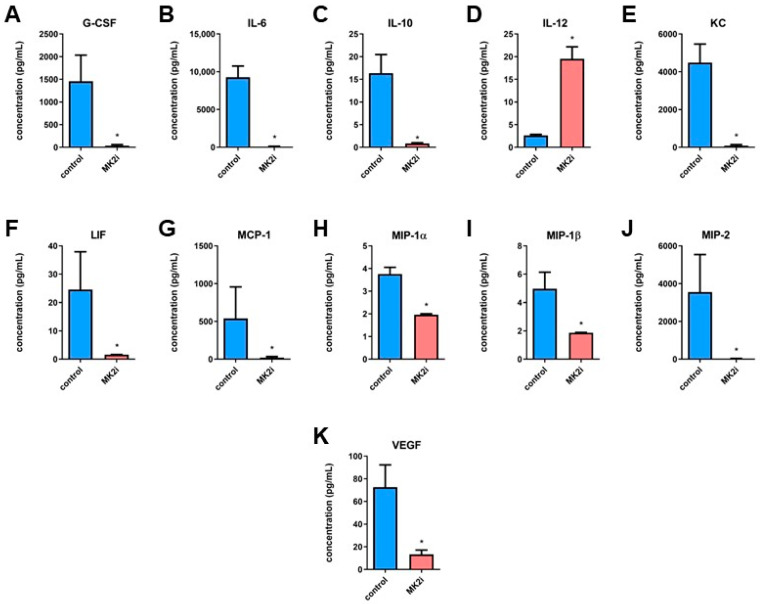
**MK2 inhibition affects production and secretion of cytokines and chemokines in PNETs.** Tumor supernatants were examined by multiplex array for the concentration of G-CSF (**A**), IL-6 (**B**), IL-10 (**C**), IL-12 (**D**), KC (**E**), LIF (**F**), MCP-1 (**G**), MIP-1α (**H**), MIP-1β (**I**), MIP-2 (**J)** and VEGF (**K**) from tumors obtained from *RipTag2* mice treated with DMSO (control, blue) and MK2 inhibitor (MK2i, red). Data are presented as means  ±  SEM; * *p* <  0.05 vs. control; n = 6 in each group.

MK2 is a potent protein kinase crucial not only for cell division and cell motility, but also may be responsible for the modulation of immune responses. Thus, we hypothesized that MK2 inhibition reduces the production and secretion of cytokines/chemokines in the PNET microenvironment. To examine this, *RipTag2* tumors were divided into 8 mg pieces and incubated for 18 h in complete media. Supernatants were collected and analyzed for cytokines/chemokines by multiplex array. Tumors exposed to MK2i showed decreased concentrations of G-CSF, IL-6, IL-10, KC, LIF, MCP-1, MIP-1α, MIP-1β and MIP-2, as well as VEGF (Figure 2A–C,E–K). To note, while the level of IL-10 was decreased, the concentration of IL-12 in tumors from animals treated with MK2i was increased compared to control (Figure 2D), which may further suggest that macrophage activity is affected by MK2 inhibition. These findings, in combination with results provided in Figure 1F, suggest that MK2 supports a pro-inflammatory microenvironment and may be involved in the macrophage-related cytokine/chemokine production that favors the progression of PNETs.

### 2.3. Inhibition of MK2 Decreases Production and Secretion of Hormones in Pancreatic Neuroendocrine Tumors

**Figure 3:**
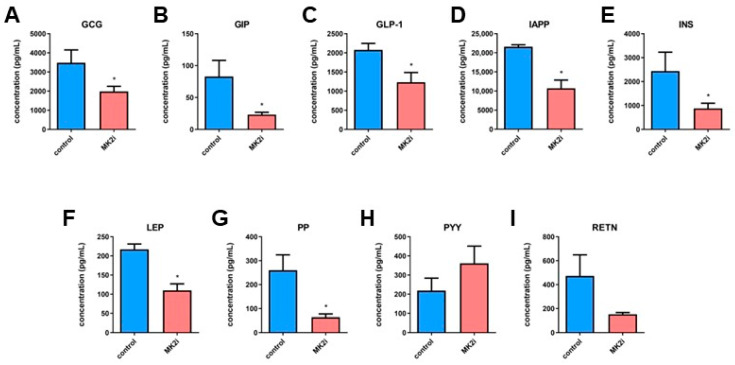
**MK2 inhibition suppress production of metabolic factors in PNETs.** Tumor supernatants were examined by multiplex array for the concentration of GCG (**A**), GIP (**B**), GLP-1 (**C**), IAPP (**D**), INS (**E**), LEP (**F**), PP (**G**), PYY (**H**) and RETN (**I**) from tumors obtained from *RipTag2* mice treated with DMSO (control, blue) and MK2 inhibitor (MK2i, red). Data are presented as means  ±  SEM; * *p* <  0.05 vs. control; n = 6 in each group.

PNETs are metabolically active tumors in mice and humans, which develop hyperinsulinemia and glucagonemia [4,16,17]. Although MK2 has not been previously linked to regulation of metabolic factors, we sought to examine the impact of MK2 inhibition in the context of the PNET metabolome. We analyzed the supernatants of PNETs obtained from the transgenic *RipTag2* mice treated with the inhibitor of MK2 compared to controls and found soluble glucagon (GCG), gastric inhibitory polypeptide (GIP), glucagon-like peptide-1 (GLP-1), amylin (IAPP), insulin (INS), leptin (LEP), pancreatic polypeptide (PP) and resistin (RETN) to be decreased (Figure 3A–G,I), while peptide YY (PYY) is slightly increased (Figure 3H). These data suggest that MK2 affected not only growth of PNETs, but also the PNET metabolome, which is implicated in tumor growth.

### 2.4. Primary PNET Cells Produce Metabolic Factors and MK2 Knockout Macrophages Show Enhanced Tumor Cell Killing

**Figure 4:**
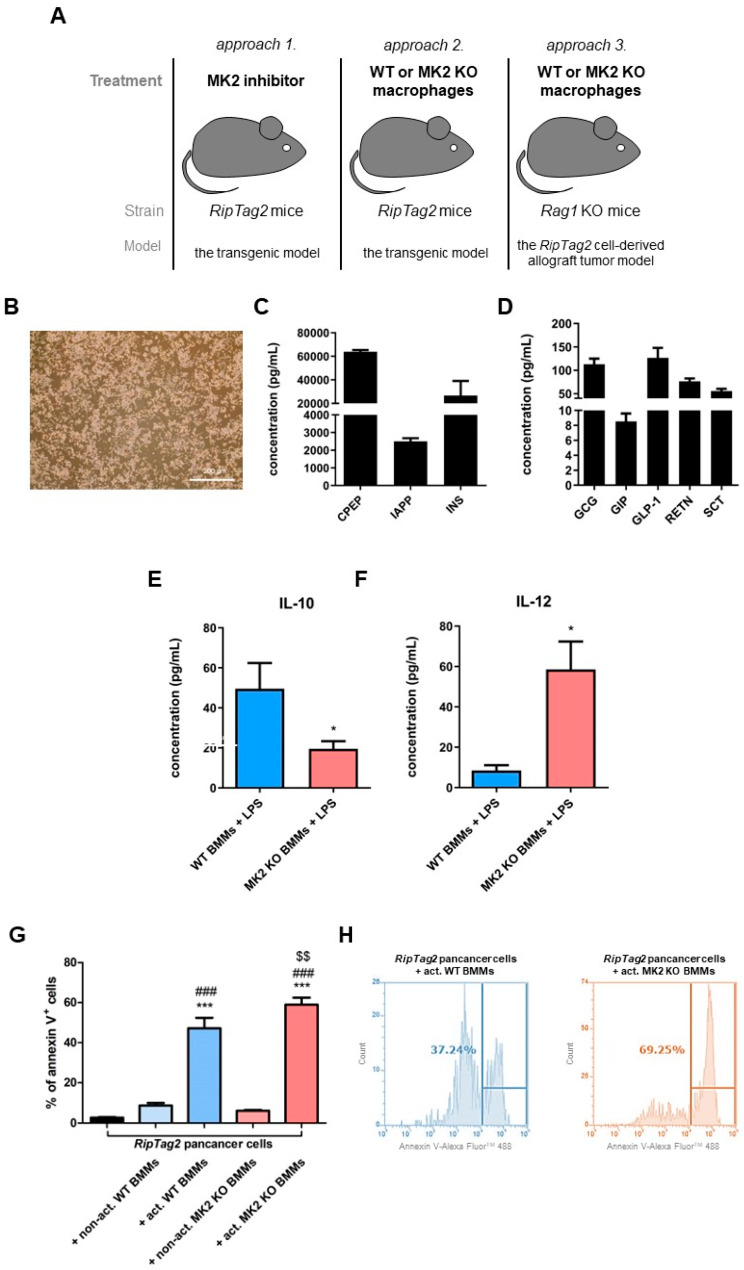
**MK2 KO BMMs are anti-tumorigenic in vitro and in vivo.** In vitro studies in the transgenic and the *RipTag2* cell-derived allograft tumor models were performed (**A**). Tumor cells (**B**) were plated and cultured from a dissociated *RipTag2* tumor. Cell supernatants were examined with a multiplex array for the concentration of CPEP, IAPP and INS (**C**), GCG, GIP, GLP-1, RETN and SCT (**D**). BMMs from WT and MK2 KO mice were cultured, and supernatants examined for the level of IL-10 (**E**) and IL-12 (**F**) by multiplex array after incubation with LPS for 24 h. The percent of annexin V^+^ cells of gated tumor cells was analyzed (**G**) in co-cultures, n = 8 in each group. Representative histograms (**H**) of the flow cytometry analysis of annexin V in the *RipTag2*cells treated with activated WT and MK2 KO BMMs. Data are presented as means  ±  SEM; * *p* <  0.05 vs. WT BMMs + LPS; *** *p* <  0.001 vs. *RipTag2* cells; ^###^ *p* <  0.001 vs. *RipTag2* cells + non. activated WT or MK2 KO BMMs; ^$$^ *p* <  0.01 vs. *RipTag2* cells + activated WT BMMs.

To further analyze the role of MK2 in PNETs, we generated the primary *RipTag2* pancreatic cancer cells from a tumor obtained from a *RipTag2* transgenic mouse (Figure 4A,B) to use in a tumor cell transfer model. We performed analysis of metabolic factors of these cells using multiplex analysis. We found that the primary *RipTag2* cells, similar to PNETs obtained from the transgenic *RipTag2* mice, were able to produce a wide spectrum of metabolic factors such as C-peptide (CPEP), GCG, GIP, GLP-1, IAPP, INS, RETN and secretin (SCT) (Figure 4C, D). To note, metabolic factors such as CPEP or SCT were detected in the primary PNET cells compared to PNETs obtained from the transgenic mice which may be related to differences in cell composition.

Since cytokine changes in tumors treated with MK2 inhibitors presented in Figure 2 suggest cytokine/chemokine changes that may be associated with the action of macrophages, cultured bone marrow-derived macrophages (BMM) were treated with LPS to examine cytokine production. We found that MK2 KO BMMs produced decreased levels of IL-10 and increased levels of IL-12 when compared to WT BMMs (Figure 4E, F), suggesting that MK2 KO BMMs have a more anti-tumorigenic phenotype than WT BMMs. IL-10 and IL-12 levels were under the detection limit in non-activated WT and MK2 KO BMMs so are not shown. In order to examine the cytotoxic potential of macrophages and the significance of MK2 KO in macrophages, a tumor killing assay was performed where WT or MK2 KO BMMs activated with LPS were incubated with primary *RipTag2* cells for 18 h. Tumor cells were stained with annexin V ^+^. As shown in Figure 4G,H, a significantly higher percent of annexin V^+^ primary *RipTag2* cells were found in co-culture of the primary *RipTag2* cells with MK2 KO BMMs when compared to cells incubated with WT BMMs, suggesting that MK2 is involved in the anti-tumorigenic action of macrophages.

### 2.5. MK2 Knockout Macrophages Improve Survival and Suppress Growth of Pancreatic Neuroendocrine Tumors

**Figure 5:**
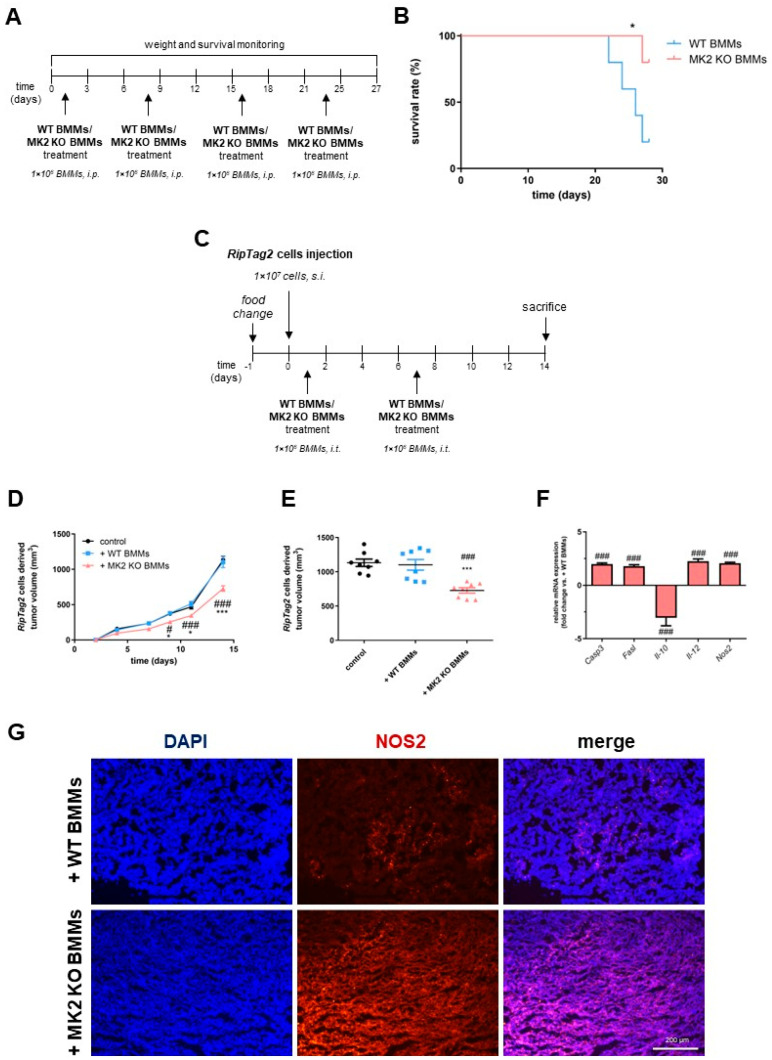
**Adoptive transfer of MK2 KO macrophages improves survival of *RipTag2* mice and suppress growth of cell line allograft tumors**. The *RipTag2* mice were treated with macrophages as shown in a timeline (**A**). The Kaplan–Meier curve (**B**) shows improved survival of mice receiving MK2 KO macrophages compared to WT macrophages, n = 8 in each group. *Rag1* KO mice receiving *RipTag2* cells were treated with macrophages as presented in the timeline (**C**), and showed decreased tumor growth (**D**, **E**) with adoptive transfer of MK2 KO macrophages, and these tumors had increased expression of apoptotic genes (**F**) such as *Casp3* and *Fasl* along with decreased expression of *Il-10* and increased expression of *Il-12* and *Nos2* as indicators of macrophage activity, n = 8 in each group. Immunofluorescence staining of NOS2 (**G**) in tumors treated with MK2 KO macrophages shows increased activity of macrophages compared to tumors treated with WT macrophages, n = 11 in each group. Data are presented as means  ±  SEM; * *p* <  0.05, *** *p* <  0.001 vs. control; ^#^ *p* <  0.05, ^###^ *p* <  0.001 vs. + WT BMMs.

To investigate the anti-tumorigenic potential of MK2 KO macrophages in the progression of PNETs, both the transgenic *RipTag2* mice and the primary *RipTag2* cell-derived allograft tumor model were employed (Figure 4A). The nine-week-old transgenic *RipTag2* mice were treated once a week with WT or MK2 KO BMMs, and weight and survival were monitored for up to four weeks (Figure 5A). As was shown in Figure 5B, the Kaplan–Meier curve indicates that *RipTag2* mice treated with MK2 KO BMMs show an improved survival rate when compared to mice treated with WT BMMs. Additionally, in our cell line model, where *Rag1* KO mice were treated with WT or MK2 KO BMMs (Figure 5C), we observed markedly suppressed tumor growth in mice treated with MK2 KO BMMs compared to animals treated with WT BMMs (Figure 5D,E). No differences were observed between male and female mouse groups for these studies. Furthermore, the cell-derived allograft tumors in mice treated with MK2 KO BMMs were characterized by significantly higher gene expression of apoptotic factors such as *Casp3* and *Fasl* when compared to mice treated with WT BMMs (Figure 5F). Moreover, in the *RipTag2* cell-derived allograft tumors of mice treated with MK2 KO BMMs, there was enhanced activity of macrophages compared to tumors of mice treated with WT BMMs, which was confirmed by increased expression of *Il-12* and *Nos2* and decreased expression of *Il-10* (Figure 5F). Immunofluorescence staining of NOS2 was also found to be higher in tumors of mice treated with MK2 KO BMMs when compared to mice treated with WT BMMs (Figure 5G). Overall, we found that MK2 affects the progression of PNETs, which is partially mediated by macrophages activity and increased apoptosis.

### 2.6. MK2 Knockout Macrophages Modulate Production and Secretion of Cytokines, Chemokines and Metabolic Factors in Pancreatic Neuroendocrine Tumors

**Figure 6:**
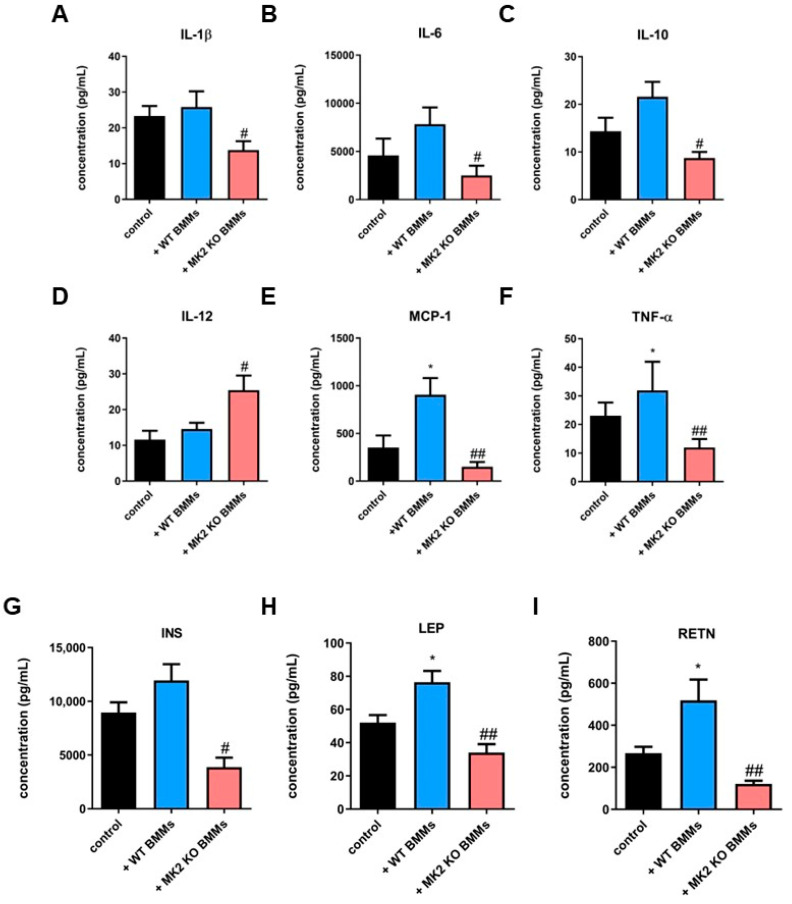
**MK2 KO macrophages inhibit production and secretion of cytokines/chemokines and metabolic factors in PNETs.** Tumor supernatants were examined by multiplex array for the concentration of IL-1β (**A**), IL-6 (**B**), IL-10 (**C**), IL-12 (**D**), MCP-1 (**E**), TNF-α (**F**), INS (**G**), LEP (**H**) and RETN (**I**) in media from the *RipTag2* cell-derived allograft tumors obtained from mice treated with PBS (control, black), WT BMMs (+WT BMMs, blue) and MK2 KO BMMs (+MK2 KO BMMs, red). Data are presented as means ± SEM; * *p* <  0.05 vs. control; ^#^ *p* <  0.05, ^##^ *p* <  0.01 vs. + WT BMMs; n = 8 in each group.

Since we found decreased tumor growth of PNETs in animals treated with MK2 KO macrophages, we next examined production and secretion of cytokines and chemokines as well as metabolic factors in PNET supernatants of the *RipTag2* cell-derived tumors obtained from mice treated with MK2 KO BMMs compared to controls and animals treated with WT BMMs. Multiplex analysis revealed higher concentration of cytokines and chemokines in the supernatants of the *RipTag2* cell-derived tumors obtained from mice treated with WT BMMs compared to controls. Significantly lower concentration of IL-1β, IL-6, IL-10, MCP-1 and TNF-α were found (Figure 6A–C,E,F), while a higher level of IL-12 was documented (Figure 6D) in the supernatants of tumors obtained from mice treated with MK2 KO BMM compared to WT BMM-treated animals.

Moreover, we show higher production of metabolic factors such as INS, LEP and RETN in the supernatants of tumors obtained from mice treated with WT BMMs compared to controls (Figure 6G–I). Further analysis indicated that the supernatants of tumors obtained from mice treated with MK2 KO BMMs compared to WT BMM-treated animals are characterized by decreased INS, LEP and RETN production/secretion (Figure 6G–I). Collectively, these data suggest that macrophages play an important role in pro-tumorigenic cytokine/chemokine production and tumor metabolism in PNETs.

## 3. Discussion

Accumulating evidence shows that pro-inflammatory cytokines in the TME favors cancer cell proliferation, expansion and survival [18,19,20]. Additionally, some studies noted that high expression of pro-inflammatory cytokines is associated with poor prognosis and worse survival of patients with various cancers [21,22]. The signaling pathways mediated by MK2 promote pro-inflammatory cytokine and chemokine production, which drives the progression of some cancers [15,23,24,25,26,27]. Nevertheless, very little is known about the TME in PNETs. In this study, we provide the first evidence that MK2 acts as a potent regulator of PNET progression where pro-tumorigenic action is associated with the production of pro-inflammatory cytokines and chemokines along with metabolic factors. The effects of MK2i were seen on a therapeutic level, where we observed that MK2i treatment improves the survival rate of the transgenic *RipTag2* mice and significantly suppresses the growth of PNETs. This action seems to be related with enhanced expression of pro-apoptotic genes such as *Casp3* and *Fasl*. On the other hand, the mechanism of action may also be associated with the blockade of pro-tumorigenic soluble factors. It should be also noted that PNETs are highly angiogenic tumors and VEGF expression correlates with patient survival, progression and metastasis in patients with PNETs [28,29]. We were able to show that MK2 inhibition affected VEGF secretion, which was drastically down-regulated after long-term administration of MK2i.

Interestingly, real-time PCR and multiplex analysis revealed changes in macrophage function in the transgenic *RipTag2* mice treated with MK2 inhibitors. In fact, administration of MK2 inhibitors to mice was manifested by higher expression of *Adgre1* (also known as F4/80), a macrophage marker. Increased expression of *Nos2* (M1 phenotype-associated marker of macrophages) and decreased expression of *Arg1* (M2 phenotype-associated marker of macrophages) as well as altered levels of macrophage-related cytokines and chemokines such as IL-10, IL-12, MCP-1, MIP-1α, MIP-1β and MIP-2 in PNETs when compared to control animals were also observed. Addition of macrophages to the PNET microenvironment led to increased cytokines and chemokines such as IL-1β, IL-6, MCP-1 and TNF-α, which are known to participate in the promotion of neoplastic transformation and the progression of tumors as well as being related to the development of immunosuppression in the TME. For instance, Kaplanov, et al., documented that the blocking IL-1β inhibits growth and reverses the immunosuppression of breast cancer in a murine model [30]. Furthermore, we and others have documented the tumor-promoting action of macrophage-related chemokine, i.e., MCP-1, which was noted in osteosarcoma to promote cancer cell migration, while we showed that MCP-1 also promotes colon cancer growth [15,31]. In contrast, IL-12, which we showed was increased by MK2 inhibition, is characterized as a major contributor of enhanced cytotoxic function in anti-tumor immunity, while IL-10 promotes tumor progression [32,33,34,35].

Our work and that of Suarez-Lopez, et al., indicate that MK2 is a potent regulator of macrophage polarization and cytokine production from macrophages characterized as a M1 phenotype in chemically induced model of colitis-associated colorectal cancer [11]. To further explore the significance of the MK2 pathway in macrophages, WT and MK2 KO BMM were examined for cytokine production in the presence of LPS, and MK2 KO BMM were found to produce decreased levels of IL-10 and increased levels of IL-12. To note, we have previously documented that MK2 activity in macrophages regulates other pro-tumorigenic chemokines [15]. Here, we also showed that MK2 KO macrophages show increased tumor cytotoxicity using primary *RipTag2* cells we generated for both in vitro and in vivo studies. These cells manifested a convergent metabolic phenotype similar to the *RipTag2* transgenic mice. Thus, the activity of MK2 seems to be related not only to regulating the growth of PNETs mediated by cytokines/chemokines, but also to the regulation of the tumor-killing ability of macrophages, suggesting that MK2 favors pro-tumorigenic phenotype of macrophages. In support of our findings, a study by Carmona-Fontaine, et al., also documented a correlation between hypoxia and the location of tumor-associated macrophages with high expression of M2 phenotype-associated markers in the transgenic *RipTag2* mice [36]. The tumor-killing effect of MK2 KO BMMs in the *RipTag2* transgenic mice may be related to a significantly better survival rate compared to animals treated with WT BMMs. Additionally, reduced tumor growth of *RipTag2* cell-derived tumors treated with MK2 KO BMMs was accompanied by increased apoptosis, reduced pro-inflammatory cytokine production and enhanced macrophage activity in the TME. Collectively, we have established that MK2 inhibition in macrophages indicate that they are at least partially responsible for the tumor-promoting activity of MK2 in PNETs.

PNETs are a heterogenous type of tumor, and patients with PNETs are characterized by differences in hormone status and numerous associated symptoms. Hormone hypersecretion is a major challenge for the promotion of PNETs, and the complications of hypersecretion may have a negative impact on diagnosis and treatment of patients [37]. Interestingly, Pan, et al., noted that metabolic signature may be a useful predictor for the evaluation of treatment efficiency in patients with neuroendocrine tumors [38]. Clinical observations highlighted that hormone hypersecretion often allows for PNET detection in earlier stages of disease, but extensive production of metabolic factors can increase the mortality of patients. In the transgenic *RipTag2* mice, metabolomic alterations have been recognized; thus, this model has proven highly instrumental for preclinical studies [39]. Our study is the first to show a direct link between MK2 signaling pathway and metabolic factor hypersecretion in PNETs. Interestingly, we found that MK2 is a metabolomic effector where MK2 inhibitor treatment was able to reduce production of GCG, GIP, GLP-1, IAPP, INS, LEP, PP or RETN in PNETs, suggesting it may be a promising therapeutic target for PNETs.

The above-mentioned metabolic factors participate in the promotion of cancer cell proliferation and the inhibition of apoptosis [40,41,42]. For example, adipokines such as LEP and RETN promote metastasis through activation of ezrin, radixin and moesin proteins in breast cancer cells [43]. Moreover, LEP and RETN affect tumor progression by promoting anti-apoptotic and angiogenic factors as well as production of pro-inflammatory cytokines [44,45,46]. Yagi, et al., found that GCG enhances colon cancer cell growth in a GCG receptor-dependent way [40]. In addition, GLP-1 is characterized by a wide spectrum of actions affecting β cell proliferation and apoptosis as well as regulation of INS and GCG secretion [47]. Indeed, some hormones may affect each other and act as a positive loop for tumor growth. An example is in breast cancer where hyperinsulinemia enhances the growth and invasive potential of breast cancer cells mediated by LEP [48]. The significance of the metabolome, especially in the context of immune responses mediated by macrophages in PNETs, has not been well investigated. Some studies indicated that macrophages exposed to hypoxia/lactate may secrete IL-6, TNF-α, CCL5 and CCL18, which favor glycolysis and boost synthesis of pro-glycogenic factors in TME in some cancers [49,50,51,52]. Furthermore, Carmona-Fontaine, et al., suggested that extracellular metabolites in the TME form gradients responsible for tumor-associated macrophage differentiation [36]. Previous studies also documented that obesity reprograms adipose tissue macrophages to a pro-inflammatory metabolically activated phenotype, and this event seems to promote triple-negative breast tumor formation in vivo [53,54]. According to a study by Kratz, et al., metabolically activated macrophages are generated by metabolic factors characteristic of metabolic syndrome such as glucose, insulin and palmitate [55]. However, PNETs are known to be a particularly metabolically active tumor type, and our analysis indicated that MK2 affects macrophage-dependent production of metabolic factors such as insulin, leptin and resistin in PNETs. Thus, this is the first study to identify MK2 as a critical regulator of metabolic factors in tumors and in macrophages in general. Collectively, our results suggest that MK2 regulates the growth of PNETs, partially in a macrophage-dependent manner, not only affecting cytokine and chemokine production, but also boosting the pro-tumorigenic function of macrophages influencing PNET metabolome.

## 4. Materials and Methods

### 4.1. Mice

Tg(RIP1-Tag)2Dh (*RipTag2*) mice were obtained from National Institute of Health (Bethesda, MD, USA), MK2^tm1Mgl^ (MK2 KO) were obtained from Dr. Mathias Gaestel (Hannover Medical School, Germany) and B6.129S7-Rag1^tm1Mom/J^ (*Rag1* KO), as well as C57BL/6 wild type (WT) mice were obtained from Jackson Laboratory (Bar Harbor, MA, USA) and bred in-house. The animals were housed at Comparative Medicine Center, University of Utah Health, UT, USA at a constant temperature (22–24 °C), relative humidity ~55%, and maintained under a 12 h light/dark cycle (lights turned on 8 a.m.) with access to standard chow pellets and water ad libitum. The University of Utah Institutional Animal Care and Use Committee approved the research protocols.

### 4.2. Bone Marrow-Derived Macrophages and MK2 Inhibition

BMMs were isolated from approximately 10-week old WT and MK2 KO mice. Briefly, femur bones were flushed with Dulbecco’s modified Eagle’s medium (DMEM, ThermoFisher Scientific, Waltham, MA, USA) under sterile conditions. Isolated cells were pelleted and resuspended in 30 mL differentiation medium, i.e., DMEM supplemented with 10% fetal bovine serum (FBS, ThermoFisher Scientific, Waltham, MA, USA), 1% L-glutamine (ThermoFisher Scientific, Waltham, MA, USA), 1% penicillin/streptomycin cocktail (Corning, Tewksbury, MA, USA) and 30% LADMAC conditioned medium. The WT and MK2 KO BMMs were incubated for seven days in order to differentiate towards the macrophage cell lineage as previously published [56,57]. WT and MK2 KO BMMs were used for in vitro and in vivo experiments.

The transgenic, *RipTag2* mice were treated as was shown in Figure 1A. Atweek eight, standard chow pellets with adjusted sucrose/corn starch diet (cat. TD.86489, Envigo, Indianapolis, IN, USA) were replaced. The male mice were treated with intraperitoneal (i.p.) injections 3 times per week with 30 µL of DMSO (control) or 10 µg of MK2 inhibitor (MK2i), i.e., PF-3644022 starting at week nine. Some *RipTag2* mice were administered (i.p.) with 1 × 10^6^ of WT or MK2 KO BMMs in PBS (Figure 5A). Control animals received equal volume of PBS as a vehicle. Mice were monitored daily for clinical parameters including body weight and mortality. PNETs were collected, weighed and divided for further analyses.

### 4.3. Isolation and Culture of Primary RipTag2 Cancer Cells

PNETs obtained from untreated 12 week old *RipTag2* mouse were extracted and dissociated mechanically in Hanks’ balanced salt solution (MilliporeSigma, Burlington, MA, USA) using gentleMACS^TM^ Dissociator (Miltenyi Biotec., Auburn, CA, USA). The cell suspension was filtrated through a 40 µm cell strainer (ThermoFisher Scientific, Waltham, MA, USA). PNET cells were cultured in Dulbecco’s modified Eagle’s medium/nutrient mixture F-12 (DMEM/F-12, ThermoFisher Scientific, Waltham, MA, USA) supplemented with 20% FBS (ThermoFisher Scientific, Waltham, MA, USA), 1% L-glutamine (ThermoFisher Scientific, Waltham, MA, USA) and 1% penicillin/streptomycin cocktail (Corning, Tewksbury, MA, USA) under standard condition for up to 24 h. The primary *RipTag2* pancreatic cancer cells were passaged up to 6 times and used for further analyses.

### 4.4. Immunofluorescence Analysis

*RipTag2* tumors were fixed in 4% paraformaldehyde for up to 24 h, blocked with 2%rat serum and incubated with commercially available antibodies against phospho-MK2 (p-MK2, cat. sc-101729, Santa Cruz Biotechnology, Santa Cruz, CA, USA) or NOS2-APC780 (cat. 47-5920-82, ThermoFisher Scientific) at 1:200 dilution. The primary *RipTag2* cancer cells were washed and incubated with donkey anti-rat secondary antibody (cat. A-21209, ThermoFisher Scientific, Waltham, MA, USA). Sections were washed and mounted in SlowFade™ Gold Antifade Mount with DAPI (cat. S36938, ThermoFisher Scientific, Waltham, MA, USA). Cells and tumors were imaged using EVOS™ M7000 Imaging System (ThermoFisher Scientific, Waltham, MA, USA).

### 4.5. Murine Allograft Model and Treatments

The primary *RipTag2* cells (1 × 10^7^) were resuspend in PBS and mixed with Matrigel^®^ (Corning, Tewksbury, MA, USA). Cells were injected into the flank of six-to-eight-week-old male and female *Rag1* KO mice. As was shown in Figure 5C, standard chow pellets with adjusted sucrose/corn starch diet were used for these mice. Mice with the *RipTag2* cell-derived allografts were administered intratumorally with 1 × 10^6^ of WT or MK2 KO BMMs resuspended in PBS. Tumor volume was measured with caliper and calculated according to the following formula: tumor size = (length × length × width)/2.

### 4.6. Tumor Killing Assay and Flow Cytometry Analysis

The primary *RipTag2* cells were plated with WT or MK2 KO BMMs in a ratio of 1:2. Some WT or MK2 KO BMMs were activated with 1µg/mL of LPS (Enzo Life Sciences, Farmingdale, NY, USA). Both non-activated and activated WT or MK2 KO BMMs werecultured with primary *RipTag2* cells and incubated for 24 h. Supernatants from co-cultures were collected for multiplex analysis. Cells were stained with F4/80 (cat. 14-4801-82, ThermoFisher Scientific, Waltham, MA, USA) and Annexin V Ready Flow Conjugate for Apoptosis Detection using Annexin Binding Buffer (cat. R37174 and cat. V13246, ThermoFisher Scientific, Waltham, MA, USA). Flow cytometry analysis was performed using an Attune^TM^ NxT Flow Cytometer and analyzed with Attune^TM^ NxT Software (ThermoFisher Scientific, Waltham, MA, USA). Macrophages were gated using F4/80 marker and the percent of annexin V^+^ cells in the population of tumor cells was calculated.

### 4.7. RNA Isolation, Reverse Transcription and Real-Time PCR

Tumor pieces were homogenized in TRIzol^TM^ reagent (cat. 15596026, ThermoFisher Scientific, Waltham, MA, USA) and RNA extraction was performed according to the manufacturer’s instructions. The quality and quantity of RNA were measured with a NanoDrop^TM^ Lite Spectrophotometer (ThermoFisher Scientific, Waltham, MA, USA). Total RNA (100 ng/µL) was reverse transcribed using High-Capacity cDNA Reverse Transcription Kit (cat. 4368814, ThermoFisher Scientific, Waltham, MA, USA) with the following PCR settings: 25 °C for 10 minutes, 37 °C for 120 minutes and 85 °C for 5 minutes. Quantitation of mRNA was performed using real-time PCR with validated FAM dye-labeled TaqMan^®^ probes (Applied Biosystems, Foster City, CA, USA) for *Actb*-Mm02619580_gL, *Adgre1*-Mm00802529_mL, *Casp3*-Mm01195085_mL, *Fasl*-Mm00438864_mL, *Il-10*-Mm01288386_mL, *Il-12*-Mm00434169_mL and *Nos2*-Mm00440502_mL. The reaction mixture consisted of cDNA, TaqMan^®^ Fast Advanced Master Mix (Applied Biosystems, Foster City, CA, USA), TaqMan^®^ Assays, and RNase-free water in a total volume of 10 µL. Cycle parameters for TaqMan^®^ assays were as follows: initial denaturation at 95 °C for 3 min, followed by 40 cycles of sequential incubations at 95 °C for 15 s and 60 °C for 1 min. Results were normalized to the expression of *Actb* gene, i.e., housekeeping gene. All experiments were performed at least as duplicates on QuantStudio™ 5 Real-Time PCR System (ThermoFisher Scientific, Waltham, MA, USA). The endpoint used in real-time PCR quantification, i.e., Ct parameter, was defined as the PCR cycle number that crossed the signal threshold. Quantification of gene expression was performed using the comparative CT method (Sequence Detector User Bulletin 2; Applied Biosystems) and reported as the fold change relative to the mRNA of the mouse housekeeping gene.

### 4.8. Multiplex Analysis

PNETs from the transgenic *RipTag2* mice and the *RipTag2* cell-derived allograft tumors were divided into 8 mg pieces (±0.5 mg) and incubated in RPMI media (Corning, Tewksbury, MA, USA), supplemented with 10% fetal bovine serum (ThermoFisher Scientific, Waltham, MA, USA), 1% penicillin/streptomycin (Corning, Tewksbury, MA, USA) and 1% L-glutamine (ThermoFisher Scientific, Waltham, MA, USA) up to 18 h. Supernatants from in vitro studies were used for multiplex arrays. The supernatants were analyzed for the levels of cytokines/chemokines and metabolic factors by multiplex arrays, mouse cytokine/chemokine panel I and mouse metabolic hormone panels (MilliporeSigma, Burlington, MA, USA) in accordance with the manufacturer’s instructions.

### 4.9. Statistical Analyses

Statistical analysis was performed using GraphPad Prism 8 (GraphPad Software Inc., San Diego, CA, USA). Results are presented as means  ±  standard error of mean (SEM). Parametric unpaired or non-parametric Mann–Whitney U tests and two-way ANOVA followed by Bonferroni’s multiple comparison post hoc test were used for comparison of studied groups. *p* values  <  0.05 was considered statistically significant.

## 5. Conclusions

Using in vitro and in vivo studies, our results indicate that MK2 may be a potent regulator of the development and the progression of PNETs. Its activity affects not only PNET cells, but also the TME of PNETs by macrophages. Our functional studies indicated that macrophages participate in tumor growth and macrophages with MK2 depletion generate similar effects as were observed with MK2 inhibitor treatment. Finally, our study highlights that MK2-mediated regulation of pro-tumorigenic activity in the TME of PNETs is complex and involves both immune responses and metabolomic factors to promote PNET growth. According to our findings, a novel treatment approach based on MK2 inhibition may be effective for both PNET growth inhibition and excess hormone elimination in PNETs in pre-clinical models. Nevertheless, it should be noted that murine models of PNETs are characterized by slightly different genetic background when compared to human PNETs and recapitulate only in part human PNETs. Further investigations need to be employed to explore translational potential of MK2 in human PNETs.

## Data Availability

The datasets generated during and/or analyzed during the current study are available from the corresponding author on reasonable request.
